# Heat-Responsive miRNAs Participate in the Regulation of Male Fertility Stability in Soybean CMS-Based F_1_ under High Temperature Stress

**DOI:** 10.3390/ijms22052446

**Published:** 2021-02-28

**Authors:** Xianlong Ding, Jinfeng Guo, Qiqi Zhang, Lifeng Yu, Tuanjie Zhao, Shouping Yang

**Affiliations:** Soybean Research Institute, National Center for Soybean Improvement, Key Laboratory of Biology and Genetic Improvement of Soybean (General, Ministry of Agriculture), State Key Laboratory of Crop Genetics and Germplasm Enhancement, Jiangsu Collaborative Innovation Center for Modern Crop Production, College of Agriculture, Nanjing Agricultural University, Nanjing 210095, China; xlding2012@163.com (X.D.); jfguo1557@163.com (J.G.); qiqizhang1030@163.com (Q.Z.); yulifeng1018@163.com (L.Y.)

**Keywords:** soybean (*Glycine max* (L.) Merr.), cytoplasmic male sterility-based F_1_, male ferility stability, high temperature stress, small RNA-sequencing, *gma-miR156b*

## Abstract

MicroRNAs (miRNAs), a class of noncoding small RNAs (sRNAs), are widely involved in the response to high temperature (HT) stress at both the seedling and flowering stages. To dissect the roles of miRNAs in regulating male fertility in soybean cytoplasmic male sterility (CMS)-based F_1_ under HT, sRNA sequencing was performed using flower buds from HT-tolerant and HT-sensitive CMS-based F_1_ combinations (NF_1_ and YF_1_, respectively). A total of 554 known miRNAs, 59 new members of known miRNAs, 712 novel miRNAs, and 1145 target genes of 580 differentially expressed miRNAs (DEMs) were identified under normal temperature and HT conditions. Further integrated analysis of sRNA and transcriptome sequencing found that 21 DEMs and 15 differentially expressed target genes, such as *gma-miR397a*/*Laccase 2*, *gma-miR399a*/*Inorganic phosphate transporter 1-4*, and *gma-miR4413a*/*PPR proteins, mitochondrial-like*, were negatively regulated under HT stress. Furthermore, all members of the gma-miR156 family were suppressed by HT stress in both NF_1_ and YF_1_, but were highly expressed in YF_1_ under HT condition. The negative correlation between *gma-miR156b* and its target gene *squamosa promoter-binding protein-like 2b* was confirmed by expression analysis, and overexpression of *gma-miR156b* in *Arabidopsis* led to male sterility under HT stress. With these results, we proposed that miRNAs play an important role in the regulation of male fertility stability in soybean CMS-based F_1_ under HT stress.

## 1. Introduction

Soybean is an important economic crop, which is one of the main sources of oil and protein. The growth and development of soybean have a certain temperature range; its reproductive period is very sensitive to temperature, especially high temperature (HT) [1]. Extreme or long-term HT has significant impacts on plant growth and reproduction [2,3,4]. The male reproductive organs of plants are more sensitive to HT than the female organs [5]. For example, stamens of tomato are more sensitive to HT than pistil [6]. Furthermore, anther indehiscence and pollen abortion caused by HT stress are widespread in many crops, such as tomato [7], cotton [8], and soybean [9,10]. HT can change the epigenetic level of plants, including methylation [11] and microRNA (miRNA) [12], to achieve their response to HT stress.

miRNAs are a class of ~21 nt noncoding small RNAs that play an important role in response to HT stress during plant reproductive development [13]. Under HT stress, plant miRNA can improve its HT tolerance by regulating flowering time, vegetative growth transition, and floral organ development [14]. For example, the miR156/157-Squamosa promoter-binding protein-like (SPL) module emerges as a pivotal regulator covering flower development, male fertility, and HT response in plants [12,15,16,17]. Recently, many known miRNAs responding to HT stress during flowering in some plants have been identified using high-throughput sequencing technology. In tomato, a total of 69 HT-responsive miRNAs were identified in small RNA (sRNA) libraries of the stamen after HT stress [6]. In rice, 8 target genes corresponding to 26 miRNAs within the four quantitative trait loci (QTL) regions were identified from panicles of HT-tolerant and HT-sensitive varieties using integrated sRNA sequencing with QTL mapping [17]. Quantitative real-time PCR (qRT-PCR) analysis confirmed that *SGT1* was negatively regulated by *miR169r-5p*. Further functional studies showed that overexpression of *miR169r-5p* increased the HT tolerance of rice at the flowering stage. In cotton, 27 and 28 differentially expressed miRNAs (DEMs) were identified from the anthers of two HT-tolerant and HT-sensitive combinations under HT stress [12,18]. It was found that miR160 and miR157 caused their overexpressed cotton plants to exhibit male sterility under HT stress by regulating the auxin signaling pathway [12].

Although many miRNAs have been found in response to HT stress during plant flowering, some miRNAs have been proven to participate in the male fertility regulation in rice and cotton under HT [6,12]. However, miRNAs associated with male fertility regulation in soybean, especially cytoplasmic male sterility (CMS)-based F_1_ under HT stress, remain largely unknown. To better understand the roles of miRNAs in regulating HT-induced male sterility in soybean, sRNA sequencing was performed using flower buds from HT-tolerant and HT-sensitive CMS-based F_1_ combinations. We found that both conserved miRNAs (miR156, miR160, miR397, miR398, miR399, etc.) and soybean-specific miRNAs (*gma-miR4413a*, etc.) responded to HT stress. Most importantly, a functional study found that *gma-miR156b*-overexpressed *Arabidopsis* plants exhibited male sterility under HT stress. Our study provided a new insight into the mechanism of male fertility stability of soybean CMS-based F_1_ under HT stress.

## 2. Results

### 2.1. Global Analysis of sRNA Sequencing Data 

**Figure 1:**
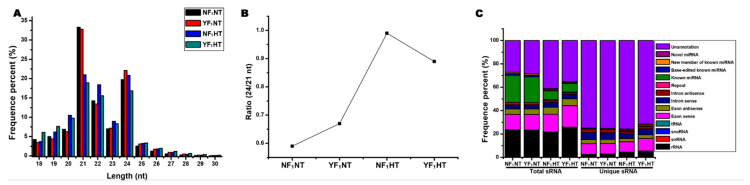
Characterization of small RNA (sRNA) in the flower bud of soybean cytoplasmic male sterility (CMS)-based F_1_ under high temperature (HT) stress. (**A**) Length distribution of sRNA sequences. (**B**) Ratio of 24/21 nt sRNAs. (**C**) Annotation of sRNA and its distribution in total sRNA and unique sRNA.

In our previous study, two soybean CMS-based hybrid F_1_ combinations, NF_1_ (HT-tolerant) and YF_1_ (HT-sensitive), were employed to explore the mechanism of male fertility stability under HT stress by RNA-Seq [9]. No difference was observed between the two soybean CMS-based F_1_ combinations under normal temperature (NT) condition [9]. After HT stress treatment, the anthers of YF_1_ were indehiscent, and the pollen fertility decreased significantly, whereas those of NF_1_ were basic normal [9]. To determine whether miRNAs were implicated in male fertility regulation under HT stress in soybean, four independent small RNA libraries were constructed using RNAs from the same samples in a previous RNA-Seq analysis [9]. An average of about 17.9 million raw reads were obtained from each sample (Table S1). Subsequently, 11.1–19.8 million clean reads were obtained after the removal of low-quality reads, adaptor reads, poly A tags, and small tags <18 nt. The average ratio of the mapping rate was above 80% (Table S1). All small RNAs (sRNAs) ranged in size from 18 to 30 nt, with high abundance at 21 and 24 nt (Figure 1A). However, both NF_1_ and YF_1_ showed the highest abundance at 21 nt, followed by 24 nt under NT (Figure 1A). There was no significant difference in the abundance of 21 and 24 nt sRNAs between the two materials under HT. However, this phenomenon changed the ratio of 24/21 nt sRNAs of both materials under NT and HT. The results showed that the ratio of 24/21 nt sRNAs under HT was higher than that under NT. NF_1_ had the highest ratio (0.99) and the lowest ratio (0.59) under HT and NT, respectively (Figure 1B). Small RNA annotation analysis showed that miRNAs accounted for 25.88% and 24.83% of the total sRNA reads in NF_1_NT and YF_1_NT, while their proportions in NF_1_HT and YF_1_HT were only 9.48% and 9.07%, respectively (Figure 1C). In general, HT stress reduces the proportion of miRNAs in total sRNA reads, while their proportion in the unique sRNA category changes in a reverse manner (Figure 1C).

### 2.2. Identification of miRNA in the Flower Bud of Soybean CMS-Based F_1_

In order to further classify miRNAs, all mappable miRNAs were compared with known miRNAs in the miRBase database (miRBase 22.0, http://www.mirbase.org/ (Access on: 12 March 2018)) to screen the known miRNA in soybean, new member of a known miRNA in soybean, and novel miRNA. A total of 554 known miRNAs belonging to 218 families were identified in all samples (Table 1 and Table S2). We found that 38 miRNA families contained more than 1 member with high abundance (transcripts per million (TPM) > 100); 10 of them have more than 10 members, such as miR156, miR164, miR166, and miR169 (Table S3). MIR156 was the family with the most members, and MIR166 was the family with highest abundance in both NF_1_ and YF_1_, making up 30.31%–93.48% TPM of these 38 miRNA families (Tables S2 and S3). In addition, 59 new members of known miRNAs belonging to 32 known miRNA families (Table S4) were detected by small RNA sequencing analysis. These 59 miRNAs have not been reported as gma-miRNAs in miRBase 22.0 previously but are homologous to known plant miRNAs. Most of them (61.02%) have a length of 21 nt, and their precursor lengths range from 72 to 353 nt. For *N-miR167a*, *N-miR167b*, and *N-miR395*, both miRNA-3p and miRNA-5p were simultaneously found on the two arms of their pre-miRNAs (Table S4). Among these new members of a known miRNA, only *N-miR395-3p*, *N-miR4345*, *N-miR4405*, *N-miR477*, and *N-miR530* were found to express in both NT and HT conditions. The remaining 48 and 7 new members of known miRNAs were identified only in NT and HT conditions, respectively (Table S4).

A total of 712 novel miRNAs belonging to 507 novel families (Table 1) were predicted in this study. Among them, there were 91 novel families with more than 1 member, such as *novel-miR028* and *novel-miR348*, with 8 and 12 members, respectively (Table S5). The precursor lengths of these novel miRNAs varied from 63 to 362 nt, with minimal folding energy indices (MFEIs) ranging from 0.86 to 3.20 (Table S5). In addition, 80 novel miRNAs (40 pairs) on both arms of the pre-miRNAs were identified. The lengths of their mature miRNAs ranged from 19 to 24 nt, and about one-third of them were 21 nt (Table S5). Most novel miRNAs are relatively low in expression (TPM < 10), and only 5 novel miRNAs were expressed in both NT and HT conditions (Table S5).

### 2.3. Identification of HT-Responsive miRNAs in the Flower Bud of Soybean CMS-Based F_1_ under HT Stress

**Figure 2:**
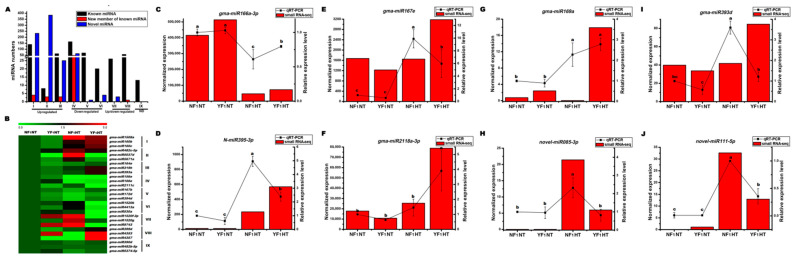
Identification and quantitative real time PCR (qRT-PCR) analysis of HT-responsive miRNAs in the flower bud of soybean CMS-based F_1_ under HT stress. (**A**,**B**) Quantity distribution and heat map of HT-responsive microRNAs (miRNAs). (**C**–**J**) qRT-PCR verification of HT-responsive miRNAs. Data are presented as means ± standard deviation (SD) from three independent biological replicates. Values with a different letter indicates statistical differences (one-way ANOVA, Duncan’s test, *p* < 0.05). The left and right *y*-axes are the expression level scales for sRNA-seq and qRT-PCR analysis, respectively.

All miRNAs were classified into nine types (types I–IX), and 36 DEMs were chosen to create a heat map according to their expression patterns (Figure 2A,B). Overall, more than 99% of the miRNAs were differentially expressed in NF_1_ and YF_1_ after HT stress (Table S6). In general, the known DEMs were basically distributed in the first eight types (types I–VIII). Interestingly, all new members of known miRNAs and novel miRNAs showed differential expression after HT stress (Figure 2A). Furthermore, all new members of known DEMs were only classified into types I, II, III, IV, and VIII, whereas the novel DEMs were mainly classified into types I–IV. Many DEMs were induced or repressed in both NF_1_ and YF_1_ after HT stress, such as *gma-miR160b* and *gma-miR166e* in types I and IV, respectively (Figure 2A,B). Some DEMs were only expressed in one sample and induced by HT stress, such as *gma-miR5037d* in type II (Figure 2B). In addition, only a few known miRNAs showed no response to HT stress, which were classified into type IX (Figure 2A).

The number and expression levels of the known miRNA and its new members were all decreased after HT stress, while the novel miRNAs behaved in a reverse manner (Table 1). Most importantly, the number and expression levels of known miRNA and its new members in YF_1_ were higher than those in NF_1_ under HT stress, indicating that known miRNA and its new members may negatively regulate the adaptive response of soybean HT-tolerant CMS-based F_1_ to HT stress (Table S6). On the contrary, the number and expression level of novel miRNA in NF_1_ were higher than those in YF_1_ under HT stress, indicating that the production of novel miRNA may be a kind of stress response to adapt to HT in HT-tolerant CMS-based F_1_. Among the DEMs, some known miRNA families (such as miR156 and miR160) exhibited the same suppressed or induced expression patterns after HT stress in both HT-tolerant and HT-sensitive soybean CMS-based F_1_ combinations (Table S6). Most importantly, they were highly expressed in one of the samples after HT stress, suggesting a conserved regulatory role of these miRNA families in HT response (Table S2). 

**Figure 3:**
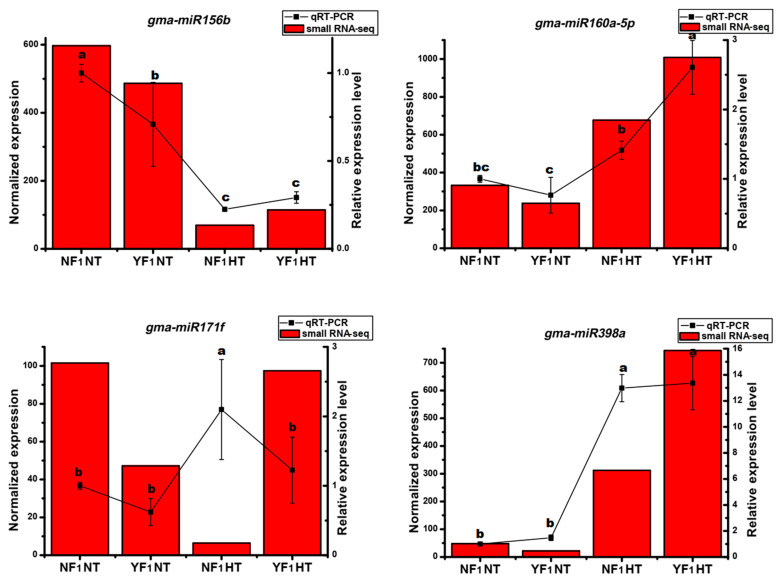
qRT-PCR analysis of HT-responsive conserved known miRNAs in the flower bud of soybean CMS-based F_1_ under HT stress. Data are presented as means ± SD from three independent biological replicates. Values with a different letter indicate statistical differences (one-way ANOVA, Duncan’s test, *p* < 0.05). The left and right *y*-axes are the expression level scales for sRNA-seq and qRT-PCR analysis, respectively.

To validate the reliability of sRNA sequencing, eight miRNAs (five known miRNAs, one new member of known miRNA, and two novel miRNAs) were selected and validated by stem-loop qRT-PCR analysis [19]. As shown in Figure 2C–J, the qRT-PCR analysis results were generally in agreement with sRNA sequencing, except for *gma-miR167e* and *N-gma-miR395-3p*, which were found to be highly expressed in NF_1_HT, but were enriched in YF_1_HT by sRNA sequencing and need further research. Many studies have reported that miR156, miR160, miR171, and miR398 are involved in heat stress response during flowering in different plant species [12,17,20]. Additionally, some members of their homologue were found to be induced or repressed by HT in this study, which was also detected by qRT-PCR analysis (Figure 3). Both sRNA sequencing and qRT-PCR found that *gma-miR156b* was repressed by HT but was highly expressed in YF_1_ under HT. The expression levels of *gma-miR160a-5p* and *gma-miR398a* were highly induced by HT in both NF_1_ and YF_1_, but more in YF_1_. However, *gma-miR171f* exhibited opposite expression changes in NF_1_HT and YF_1_HT by sRNA sequencing and qRT-PCR analysis, and needs further research.

### 2.4. Target Prediction of DEMs in the Flower Bud of Soybean CMS-Based F_1_

A total of 1145 target genes of 316 known DEMs, 30 new members of known DEMs, and 234 novel DEMs were predicted using the miRNA target gene prediction tool (Table S7). Most importantly, 129 target genes (18.8% of target genes of known DEMs) were also found to be cleaved by 143 known DEMs using degradome analysis in our previous study [21]. Many target genes encode transcription factors (TFs), including SPL, GAMYB-like, auxin response factor (ARF), NAM/ATAF/CUC (NAC), homeodomain–leucine zipper (HD-ZIP), nuclear transcription factor Y subunit A (NF-YA), GRAS, APETALA 2, and Teosinte-branched 1/Cycloidea/Proliferating (TCP). Some of these TFs are involved in the regulation of flower development or plant hormone signal transduction; for example, SPL2 controls floral organ development [16], and ARF participates in auxin signaling to modulate male fertility [12,22], which were target genes of miR156 and miR160, respectively. Some other target genes were annotated as pentatricopeptide repeat (PPR) protein and laccase (LAC), which are both associated with male fertility regulation [23,24,25]. All these four types of target genes were also detected by both the miRNA target prediction tool in this study and degradome sequencing from our previous study (Table S7) [21].

### 2.5. Integrated Analysis of DEMs and Differentially Expressed Genes in the Flower Bud of Soybean CMS-Based F_1_ under HT Stress

**Figure 4:**
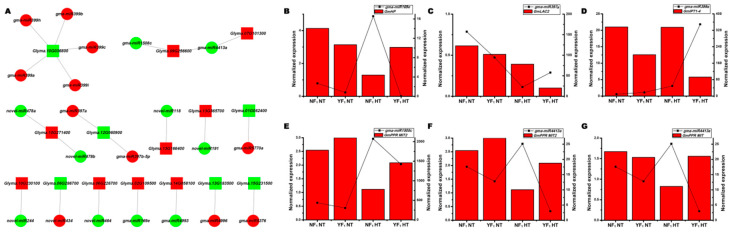
Regulatory network of miRNA-target in the flower bud of soybean CMS-based F_1_ under HT stress. (**A**) Regulatory network of miRNA-target under HT condition. Red/green circle/box indicates up or downregulated miRNA/target, respectively. (**B**–**G**) Expression levels of HT-responsive miRNAs and their targets in the flower bud of soybean CMS-based F_1_ under HT stress. The right and left *y*-axes are the normalized expression level scales for miRNA and its target, respectively.

To better understand the function of DEMs in the flower bud development of soybean CMS-based F_1_ under HT stress, all predicted target genes of DEMs were further searched and aligned against the differentially expressed genes (DEGs) identified from our previous transcriptome sequencing data of the same samples [9]. Results showed that 21 DEMs (15 miRNA families) and 15 DEGs were negatively regulated under HT stress (Figure 4A). gma-miR397 and *gma-miR399a* were induced by HT in YF_1_, and their targets, *LAC2* (*Glyma.12G060900*) and *inorganic phosphate transporter 1-4* (*GmIPT1-4, Glyma.10G036800*), were inhibited in the flower bud of YF_1_ under HT stress. On the contrary, *gma-miR169e* was induced by HT in NF_1_, and its target, a hypothetical protein (*HP, Glyma.02G109500*), was significantly inhibited under HT condition. Interestingly, the differentially expressed PPR gene (*Glyma.09G256600*) was targeted by the differentially expressed *gma-miR1508c* and *gma-miR4413a* simultaneously (Figure 4A). Most importantly, they have a negative regulatory relationship under both NT and HT conditions (Figure 4E,F). In addition, some novel DEMs also exhibited negative correlation expression patterns with their target genes according to transcription analysis (Figure 4A).

### 2.6. Overexpression of gma-miR156b Decreased Male Fertility in Arabidopsis under HT Stress

**Figure 5:**
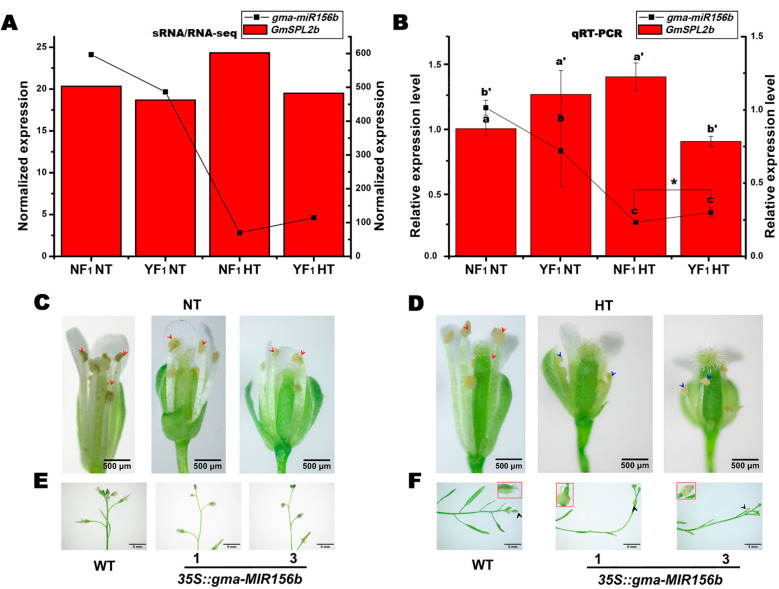
Effect of long-term moderate HT on male fertility in wild-type (WT) and *35S::gma-MIR156b* lines of *Arabidopsis.* (**A**,**B**) Expression levels of *gma-miR156b* and its target *GmSPL2b* in sRNA/RNA sequencing and qRT-PCR analysis. Data are presented as means ± SD from three independent biological replicates. Values with a different letter indicate statistical differences (one-way ANOVA, Duncan’s test, *p* < 0.05). Student’s *t*-test was performed to compare the *gma-miR156b* expression difference between NF_1_HT and YF_1_HT. Asterisk indicates statistical differences, * *p* < 0.05. The left and right *y*-axes are the expression level scales for *GmSPL2b* and *gma-miR156b*, respectively. (**C**,**D**) Phenotype of anthers in WT and *35S::gma-MIR156b* lines under long-term moderate HT condition. The red and blue arrows indicate dehiscent and indehiscent anthers, respectively. Scale bars = 500 µm. (**E**,**F**) Phenotype of inflorescence in WT and *35S::gma-MIR156b* lines under long-term moderate HT condition. The black arrow indicates yellowed and wilted flower under moderate HT condition. The flower in the red box in the upper-right/left corner is a magnified view of the flower pointed by the black arrow. Scale bars = 5 mm..

**Figure 6:**
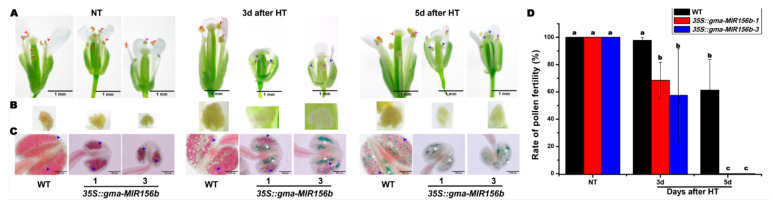
Effect of short-term extremely HT on male fertility in WT and *35S::gma-MIR156b* lines of *Arabidopsis.* (**A**) Phenotype of flowers in WT and 35S::*gma-MIR156b* lines after short-term extremely HT stress. The red and blue arrows indicate dehiscent and indehiscent anthers, respectively. Scale bars = 1 mm. (**B**) Phenotype of anthers in WT and *35S:*:*gma-MIR156b* lines after short-term extremely HT stress. An enlarged view of the anthers in (**A**) indicated by pink arrows is shown. (**C**) Pollen fertility in WT and *35S::gma-MIR156b* lines after short-term extremely HT stress. The red and green pollens pointed by blue and white arrows indicate fertile and sterile pollens, respectively. Scale bars = 100 µm. (**D**) Calculation of pollen fertility rate in WT and *35S::gma-MIR156b* lines after short-term extremely HT stress (*n* = 10). Data are presented as means ± SD from independent biological replicates. Values with a different letter indicates statistical differences (one-way ANOVA, Duncan’s test, *p* < 0.05).

The miR156/SPL network has been proven to improve the tolerance of *Arabidopsis* and alfalfa seedlings to HT [26,27], but its role under HT stress during plant flowering remains unknown. According to the sRNA sequencing analysis, gma-miR156 was suppressed by HT but was highly expressed in the flower bud of HT-sensitive CMS-based F_1_ (Table S6, Figure 5). Most importantly, the negative correlation between *gma-miR156b* and its target gene *GmSPL2b* under HT was confirmed by sequencing data and qRT-PCR analysis (Figure 5). Furthermore, our previous study found that *GmSPL2b* could be successfully cleaved by *gma-miR156b* using degradome analysis and 5′-RNA ligase mediated rapid amplification of cDNA ends (RLM-5′-RACE) assay [28]. To identify the role of *gma-miR156b* in the response to HT stress during flowering, two *35S::gma-MIR156b* transgenic *Arabidopsis* lines constitutively overexpressing the precursor of *gma-miR156b* from our previous study were used for HT treatment, and different HT conditions (30 and 42 °C) from hours to days were performed. There was no significant difference in anther dehiscence and pollen fertility between *35S::gma-MIR156b* transgenic *Arabidopsis* lines and wild-type (WT) plants under NT condition (Figure 5 and Figure 6). After 3 days of moderate HT treatment, the *gma-miR156b*-overexpressed *Arabidopsis* lines showed anther indehiscence (Figure 5D). In addition, we found some other HT damage phenomena, such as yellowing and wilting of some flowers in *gma-miR156b*-overexpressed *Arabidopsis* lines under HT stress (Figure 5F). However, the WT showed to be normal under moderate HT stress (Figure 5E).

For a better comparison of male fertility changes between *35S::gma-MIR156b* transgenic *Arabidopsis* lines and WT plants under HT, extremely HT treatment was adopted at 42 °C for 4 h. As shown in Figure 6, anther indehiscence of the *gma-miR156b*-overexpressed *Arabidopsis* lines was observed on the 3rd day after HT stress, and the pollen fertility ranged from 3.75% to 91.67% (with an average of 62.89%), which was significantly lower than that of WT plants (with an average of 97.70%). Importantly, the *gma-miR156b*-overexpressed *Arabidopsis* lines showed anther indehiscence with little and completely sterile pollen on the 5th day after HT stress (Figure 6C). One the other hand, the WT showed anther dehiscence but partial pollen abortion. All these results showed that the transgenic plants were more sensitive to HT stress.

## 3. Discussion

### 3.1. A Complex Regulatory Network of sRNAs Exists in the Flower Bud Development of Soybean CMS-Based F_1_ under HT Stress

In the past few years, many epigenetic regulators, including sRNA and DNA methylation, have been involved in plant reproductive development regulation under HT stress [11,12,18]. SiRNA and miRNA are two classes of ~24 and 21 nt sRNAs in plant, respectively [29]. Our results showed that both types of sRNAs are enriched in the flower bud of soybean CMS-based F_1_ (Figure 1A). Like sRNAs in cotton, the distribution and expression changes of sRNAs were also different in our study, whether before or after HT or between HT-tolerant and HT-sensitive CMS-based F_1_. Unlike sRNAs in many crops, the highest abundance of 21 nt sRNA was found in this study (Figure 1A), which is also not within the typical sRNA length distribution for the female parent [30]. This interesting phenomenon may be due to the hybrid F_1_ materials used in this study. The same results were found in *Brassica* and *Spartina* F_1_ hybrids, whose relative amount of ~21 nt sRNAs are more predominant than their parents’ [31,32]. In this situation, there may be other epigenetic regulators also involved in the developmental regulation of F_1_ hybrid, such as siRNA and DNA methylation.

In previous studies, it was found that the ratio of 24/21 nt sRNAs increased during anther development in cotton after HT stress, and the change of DNA methylation was that 24 nt siRNA was involved in the RNA-directed DNA methylation [12,18,33,34]. In this study, the ratio of 24/21 nt sRNAs also increased after HT stress, indicating that both siRNA and DNA methylation were involved in the regulation of flower bud development in soybean CMS-based F_1_ under HT, and DNA methylation may be increased after HT. Furthermore, recent studies also uncovered that the increased frequency of DNA methylation inhibits the expression of miRNAs [34,35]. In this study, it was found that after HT treatment, the number and expression levels of known miRNAs (except novel miRNAs) decreased, and they were also lower in NF_1_ compared with those in YF_1_ under HT condition, which corresponded to the change of 24/21 nt sRNA ratio, sRNA annotation, and mapping results (Figure 1, Table S1). A lower ratio of 24/21 nt sRNAs in YF_1_HT indicated that more miRNAs were involved in the post-transcriptional regulation of male fertility in YF_1_ under HT. The increase in the number and expression levels of novel miRNAs in NF_1_ after HT stress may be attributed to the adaptation to HT, thus maintaining the stability of male fertility under HT condition. All these results showed that a complex regulatory network of sRNAs exists in the flower bud development of soybean CMS-based F_1_ under HT, and miRNA might play an important role in the response to HT stress. However, the relationship between sRNA and DNA methylation in soybean during flower bud development under HT stress needs further study.

### 3.2. Heat-Responsive miRNAs Involved in the Regulation of Male Fertility under HT Stress

In this study, most identified miRNAs were differentially expressed under HT stress, indicating that miRNAs are involved in the regulation of male fertility and response to HT. However, the expression levels of known miRNAs (including new member of known miRNA) were repressed by HT, which is consistent with a research in rice that showed that most differentially expressed known miRNAs were downregulated during flowering under HT stress [17]. Many conserved DEM families, such as miR156, miR160, miR171, miR397, and miR398, show a response to HT stress at both the seedling and flowering stages in many plants [12,17,20,36,37,38,39,40]. These results indicate that these miRNAs play an important role in the plant response to HT stress. However, their expression trends and functions under HT are very complex at different growth stages. For example, miR160 showed high abundance expression in *Arabidopsis* and cotton at both the seedling and flowering stages after HT stress, respectively [12,41]. Furthermore, overexpression of miR160 in *Arabidopsis* increased the survival rate of seedlings under HT stress [41], whereas its overexpression in cotton inhibited the expression of *ARF10* and *ARF17*, leading to anther indehiscence [12]. In this study, all five members of miR160 were induced by HT (Table S2). Most importantly, they were more highly expressed in YF_1_HT than in NF_1_HT. Therefore, miR160 might also participate in the regulation of anther dehiscence of soybean HT-sensitive CMS-based F_1_ under HT stress.

miR397 was inhibited by HT at the seedling stage in *Arabidopsis* [38], cassava [42], and *Oryza sativa* [43], and it was also downregulated at the flowering stage in rice [17] and in our study under HT condition (Figure 4). In our study, *GmLAC2* was negatively correlated with gma-miR397, suggesting that the miR397/*LAC2* pathway might be tightly linked to the HT stress response signal in the flower bud of soybean CMS-based F_1_.

Interestingly, some miRNAs showed an opposite expression trend under HT stress at the seedling and flowering stages. For example, miR398 is induced by HT at the seedling stage in rice [43], Chinese cabbage [39], *Populus tomentosa* [44], and tomato [40], whereas it is shown to be downregulated under HT stress during flowering in rice [17,20] and wheat [45]. Most importantly, it is upregulated in HT-sensitive rice under HT when compared with HT-tolerant material [17]. The same result was found in this study; *gma-miR398a* and gma-miR398b were induced by HT and upregulated in the flower bud of HT-sensitive CMS-based F_1_ (Table S1, Figure 3).

miR399 is another conserved miRNA and plays a crucial role in regulating both the Pi homeostasis and reproductive development of the plant [46,47]. In this study, four members of miR399 (a, b, c, and h) were found to be lowly expressed in the flower bud of HT-tolerant and HT-sensitive CMS-based F_1_ under NT, and their expression patterns were similar to those of *gma-miR398a* and *gma-miR398b* after HT stress (Table S1, Figure 3). Previous studies have demonstrated that miR399-*PHO2*/*UBC24* modules affect flowering time in response to ambient temperature changes and male fertility in *Arabidopsis* and *Citrus* [47,48], respectively. *gma-miR399a* was negatively correlated with *GmIPT1-4* under HT (Figure 4), and *GmIPT1-4* (*Glyma.10G036800*) was found to be highly expressed in the flower of soybean (Phytozome v12.1, https:// phytozome.jgi.doe.gov/pz/portal.html# accessed on 14 February 2021), suggesting that the *gma-miR399a*/*GmIPT1-4* module might mediate the fertility regulation of soybean CMS-based F_1_ under HT stress.

In addition to conserved miRNAs, many nonconserved miRNAs were also found in response to HT stress, such as *gma-miR4413a* (Table S3). *gma-miR4413a* is a soybean-specific miRNA, and sRNA sequencing data showed that *gma-miR4413a* and its target PPR proteins, mitochondrial-like (*PPR MIT*, *Glyma.07G101300*, and *Glyma.09G256600*), responded to HT stress and were negatively regulated under HT condition (Figure 4). The PPR protein is encoded in the nuclear genome and has been proven to play a central role in plant C-to-U RNA editing [49,50]. RNA editing is a post-transcriptional process and enriched in organelle (chloroplast and mitochondrial) genes, which play an important role in adaptation to environmental changes, such as HT stress [51,52,53]. Recent studies found that the C-to-U editing level of grape and *Arabidopsis* decreased significantly under HT stress [51,53]. Most importantly, the expression level of most PPR genes decreased with the temperature rise, which is consistent with the reduction of C-to-U editing rates under HT [51]. Our result showed that HT induced the expression of *gma-miR4413a* in HT-tolerant CMS-based F_1_, leading to a decrease in *GmPPR MIT* expression, which may reduce mitochondrial RNA editing level and ultimately promote HT stress adaptation. However, whether RNA editing events are involved in male fertility regulation in soybean CMS-based F_1_ is unclear, and the *gma-miR4413a*/*GmPPR MIT* network with RNA editing remains to be further clarified.

### 3.3. miR156 Plays an Important Role in the Regulation of Male Fertility under HT Stress

miR156 is one of the most conserved miRNA families in plant [16]. It plays an important role in adaptation to HT stress, but is inhibited by HT stress during flowering in many plants, such as rice [17,20], cotton [12], and tomato [6]. Although HT inhibits the expression of miR156 during flowering in rice, it is highly expressed in HT-sensitive material compared with an HT-tolerant line [17]. The *Arabidopsis cngc16* mutant is very sensitive to HT and has exhibited male sterility under HT; a further study showed that HT induced the upregulation of miR156 in the mutant [54]. miR157 is also one of the highly conserved miRNA families in plant and is highly homologous to miR156. In addition, the targets of both miRNAs are SPL [55]. Moreover, the overexpression of miR157 induced the sensitivity of cotton anthers to HT stress, which showed pollen abortion and anther indehiscence under HT condition [12].

Our sRNA sequencing data showed that the expression of all 25 miR156 family members was inhibited by HT in soybean, but they were highly expressed in HT-sensitive CMS-based F_1_. qRT-PCR analysis found that *gma-miR156b* was negatively correlated with its predicted target *GmSPL2b* (Figure 5). Additionally, our previous degradome and RLM-5′-RACE analyses showed that *gma-miR156b* directed the cleavage of the *GmSPL2b* transcript [28]. Our previous research also showed that *gma-miR156b* from soybean CMS mediated the floral organ development in *Arabidopsis* [28]. In this study, we found that HT stress induced the sensitivity of male fertility in *gma-miR156b*-overexpressed *Arabidopsis* lines (Figure 5 and Figure 6). It was found in *Arabidopsis* that miR156/SPL2 controlled male fertility in plant and SPL2 partially restored male fertility in the spl8 mutant [15,16]. All these results reveal that the miR156/SPL2 module plays an important role in the regulation of plant male fertility under both NT and HT stress conditions. However, the relationship between the *gma-miR156b*/*GmSPL2b* module and the stability of male fertility in soybean CMS-based F_1_ under HT remains unclear and needs more studies.

## 4. Materials and Methods

### 4.1. Plant Materials, HT Treatment, and Sample Collection

Soybean HT-tolerant CMS-based F_1_ (NF_1_) and HT-sensitive CMS-based F_1_ (YF_1_) were used for sRNA sequencing [9]. Growth conditions and HT treatment for soybean CMS-based F_1_ combinations were performed as described previously [9]. Seedling plants were grown in illuminated incubators (RXZ-430D, Ningbo Jiangnan, Ningbo, China) at 26 ± 1/20 ± 1 °C (day/night) with a 12 h light/12 h dark photoperiod. The flowering plants (R1 stage) were grown at 30 ± 1/24 ± 1 °C (day/night) with a 12 h light/12 h dark photoperiod illuminated incubator. For HT treatment, the flowering plants were incubated at 38 ± 1/32 ± 1 °C (day/night) in the illuminated incubator with the same light condition as NT. Flower buds of each genotype (NT and HT on NF_1_ and YF_1_ genotypes) were collected from three individual plants, which were the same as the previous transcriptome sequencing samples [9].

Two *gma-miR156b*-overexpressed *Arabidopsis* lines and WT (Columbia, Col-0) were used for a functional study of *gma-miR156b* under HT stress [28]. All of the *Arabidopsis* plants were grown at 23 °C with long day conditions (16 h light/8 h dark) in an illuminated incubator (RXZ-430D, Ningbo Jiangnan, Ningbo, China). For long-term moderate HT treatment, both *gma-miR156b*-overexpressed *Arabidopsis* lines and WT were transferred into an illuminated incubator at 30 °C with 75% relative humidity (RH) for 5 days. Anther dehiscence and inflorescence growth were observed during this period. For short-term extremely HT treatment, all plants were shifted from 23 °C to 42 °C as described previously [9], and then exposed to 42 °C at 75% RH for 4 h. Anther dehiscence and pollen fertility were observed during the 5 days of restoration at 23 °C. All types were grown at 23 °C as NT condition.

### 4.2. Small RNA Sequencing Library Construction and Bioinformatics Analysis

Total RNAs were extracted using the TRIzol reagent (Invitrogen, Carlsbad, CA, United States) according to the manufacturer’s protocol. Each sRNA library was composed of a mixture of three individual plants of the corresponding material with the same amount of RNA. Four small RNA libraries were constructed and sequenced by Gene Denovo Biotechnology Co. (Guangzhou, China) using the Illumina HiSeq^TM^ 2500 System. After sequencing, raw sequencing reads were processed into clean reads by filtering out adapters or low-quality bases. All clean tags were aligned with small RNAs in the GeneBank database (Release 209.0) and Rfam (v 11.0) to identify and remove rRNA, scRNA, snoRNA, snRNA, and tRNA. Next, all of the clean tags were also aligned with the soybean reference genome (Wm82.a2.v1). Those mapped to exons or introns might be fragments from mRNA degradation, so these tags were removed. The tags mapped to repeat sequences were also removed. Then, to identify known miRNAs, clean reads were compared with miRNAs in soybean deposited at miRBase 22.0 (http://www.mirbase.org/) (Access on: 12 March 2018). The miRNA sequences of soybean were still not included in miRBase 22.0. For these miRNAs, their alignment with miRNAs from other species is a dependable way to identify the new members of a known miRNA in soybean. Finally, other sequences not mapped to known miRNAs were aligned with the reference genome, known as unannotated sequences, for novel miRNA prediction. The datasets generated by this study can be found in the NCBI using accession number PRJNA700836.

Potentially novel miRNAs were identified using MIREAP (v0.2) (https://source- forge.net/projects/mireap/) (Access on: 27 March 2013), and their secondary structures were predicted by the mfold web server (http://mfold.rna.albany.edu/?q=mfold/RNA-Folding-Form) [56]. The criteria used for selecting novel miRNAs must meet the following five characteristics as described previously [21,30]: (1) the candidate miRNA-5p and miRNA-3p are derived from opposite stem arms with minimal matched nucleotide pairs exceeding 16 nt and with maximal size differences of up to 4 nt; (2) the most abundant reads from each arm of the precursor must pair in the mature miRNA duplex with a 2-nt 3ʹoverhang; (3) the number of asymmetric bulges within the miRNA-5p/miRNA-3p duplex must be one or fewer, and the size of the asymmetric bulges must be two bases or smaller; (4) the miRNA-5p or miRNA-3p must be no more than 10 reads in one of the samples; (5) and the candidate miRNA precursor must have high negative minimal folding energy (MFE) and MFEI, with MFE <−0.2 kcal/mol/nt and MFEI >0.85.

Total miRNA consists of a known miRNA, a new member of a known miRNA, and a novel miRNA. Based on their expression in each sample, the miRNA expression level was calculated and normalized to TPM. The formula is as follows: TPM = (actual miRNA counts/total counts of clean tags) × 10^6^. The identified miRNAs had a fold change ≥2 and *p*-value < 0.05 in comparison with significant DEMs.

### 4.3. Target Gene Prediction

The software PatMatch (v1.2) was used to predict target genes of miRNA. The default parameters were as follows: (1) no more than 4 mismatches between miRNA and target (G-U bases count as 0.5 mismatches), (2) no more than 2 adjacent mismatches in the miRNA/target duplex, (3) no adjacent mismatches in positions 2–12 of the miRNA/target duplex (5’ of miRNA), (4) no mismatches in positions 10–11 of the miRNA/target duplex, (5) no more than 2.5 mismatches in positions 1–12 of the miRNA/target duplex (5’ of miRNA), and (6) MFE of the miRNA/target duplex ≥75% of the MFE of the miRNA bound to its perfect complement. According to Chinese cabbage CMS [57] and our previous study [21], a cutoff of >1.5-fold change was used for the integrated analysis of DEMs and DEGs in the flower bud of soybean CMS-based F_1_ under HT stress.

### 4.4. Inflorescence and Male Fertility Observation

The observation of anther dehiscence and inflorescence growth was performed under an Olympus SZ61 microscope (Olympus, Tokyo, Japan) with a digital color camera system (Olympus DP27, Olympus, Tokyo, Japan). The analysis of pollen fertility was performed using Alexander’s staining as described by Li [58], and the stained anthers were observed using an Olympus CX31 microscope (Olympus, Tokyo, Japan) and photographed with a digital color camera system (Olympus DP27, Olympus, Tokyo, Japan). One-way ANOVA and Duncan’s test were performed for statistical analysis.

### 4.5. qRT-PCR Analysis

Stem-loop qRT-PCR [19] and normal qRT-PCR analysis were carried out to validate the differential expression levels of miRNAs and mRNAs, respectively. All primers (Table S8) were designed based on the mature miRNA and mRNA sequences and synthesized commercially (General Biosystems, Chuzhou, China). Total RNAs from the same soybean samples that constructed the sRNA library were used for qRT-PCR analysis. According to the procedures provided in the miRNA 1st-Strand cDNA Synthesis Kit (Vazyme, Nanjing, China) and HiScript Q RT SuperMix for qPCR kit (+gDNA wiper, Vazyme, Nanjing, China), 1 µg of total RNA was reverse-transcribed using the stem-loop primer and oligo (dT) primer, respectively. The miRNA and mRNA qRT-PCR analysis was carried out using miRNA Universal SYBR qPCR Master Mix (Vazyme, Nanjing, China) and AceQ qPCR SYBR Green Master Mix (Vazyme, Nanjing, China) on a Bio-Rad CFX96 instrument (CFX96 Touch, Bio-Rad, Hercules, CA, USA), respectively. All reactions were run with three independent biological replicates, and *gma-miR1520d* [59] was used as an internal control gene for soybean. The control setup for qRT-PCR analysis of soybean was as previously described [9]. The relative expression levels of the genes were quantified using the 2^−ΔΔCt^ method [60]. Significant differences were evaluated using one-way ANOVA and Duncan’s test or Student’s *t*-test.

## Figures and Tables

**Table 1:** Data statistics of miRNA in the flower bud of soybean CMS-based F_1_ under HT stress.

Type		1. Total Number				Family Number	2. TPM ^(a)^			
	Total	NF_1_NT	YF_1_NT	NF_1_HT	YF_1_HT		NF_1_NT	YF_1_NT	NF_1_HT	YF_1_HT
Known miRNA	554 (100%)	509 (91.88%)	490 (88.45%)	375 (67.69%)	409 (73.83%)	218	8505464.44	9324598.12	2868203.98	3166645.74
New member of known miRNA	59 (100%)	52 (88.14%)	52 (88.14%)	9 (15.25%)	8 (13.56%)	32	5363.10	6283.07	666.80	961.31
Novel miRNA	712 (100%)	67 (9.41%)	72 (10.11%)	616 (86.52%)	259 (36.38%)	507	1169.21	991.34	11806.20	7669.01

(a) TPM: tags per million. Total TPM for each type of the miRNAs.

## Data Availability

The raw data of the sRNA-Seq have been submitted to NCBI Sequence Read Archive (SRA) under BioProject accession PRJNA700836.

## Supplementary Materials

Supplementary materials can be found at https://www.mdpi.com/1422-0067/22/5/2446/s1, Table S1: Data statistics of small RNA libraries in flower bud of soybean CMS-based F_1_ under HT stress; Table S2: Identification of known miRNAs in flower bud of soybean CMS-based F_1_ under HT stress; Table S3: Identification of known miRNA families with highly abundant in flower bud of soybean CMS-based F_1_ under HT stress; Table S4: Identification of new members of known miRNA family in flower bud of soybean CMS-based F_1_ under HT stress; Table S5: Identification of novel miRNAs in flower bud of soybean CMS-based F_1_ under HT stress; Table S6: Identification of HT-responsive miRNAs in flower bud of soybean CMS-based F_1_ under HT stress; Table S7: The predicted targets of miRNAs identified in flower bud of soybean CMS-based F_1_ under HT stress; Table S8: Primers used in this study.

## Abbreviations

ARF	auxin response factor
CMS	cytoplasmic male sterility
DEG	differentially expressed gene
DEM	differentially expressed miRNA
HD-ZIP	homeodomain–leucine zipper
HT	high temperature
IPT	inorganic phosphate transporter
LAC	laccase
MFE	minimal folding energy
MFEIs	minimal folding energy indices
miRNA	microRNA
NAC	NAM/ATAF/CUC
NT	normal temperature
NF-YA	nuclear transcription factor Y subunit A
PPR	pentatricopeptide repeat
PPR MIT	PPR proteins, mitochondrial-like
qRT-PCR	quantitative real-time PCR
QTL	quantitative trait loci
RH	relative humidity
RLM-5′-RACE	5′-RNA ligase mediated rapid amplification of cDNA ends
SD	standard deviation
sRNA	small RNA
SPL	Squamosa promoter-binding protein-like
TCP	Teosinte-branched 1/Cycloidea/Proliferating
TF	transcription factor
TPM	transcripts per million
WT	wild-type
